# Electrochemical Biosensor-Based Evaluation of Cholinergic Biomarkers in Experimental Nephropathy

**DOI:** 10.1021/acs.analchem.6c01440

**Published:** 2026-07-12

**Authors:** Marius Butkevicius, Ieva Sakinyte-Urbikiene, Igor Seniuk, Liubov Galuzinska, Dmytro Lytkin, Julija Razumiene

**Affiliations:** † Department of Bioanalysis, 54694 Institute of Biochemistry, Life Sciences Center Vilnius University, Sauletekio Av. 7, Vilnius LT- 10257, Lithuania; ‡ National University of Pharmacy, Ministry of Health of Ukraine, Hryhoriia Skovorody Str, 53, Kharkiv 61002, Ukraine

## Abstract

This study presents a rapid method for evaluating cholinesterase (ChE) activity using an amperometric choline biosensor based on an enzymatic membrane incorporating choline oxidase (ChOx) and a selectivity-providing acetylated cellulose layer. The developed biosensor shows a linear response range of 5–500 μM for choline with a sensitivity of 32.2 ± 0.3 μA/(mM cm^2^), enabling the determination of choline in serum. The biosensor was also adapted for acetylcholinesterase (AChE) and butyrylcholinesterase (BChE) determination, achieving LODs of 0.003 and 0.005 U/ml, respectively, with a linear range up to 0.5 U/ml. Notably, the biosensor retains 88% of its initial sensitivity after 28 days of use. Two experimental rat nephropathy models were investigated: mild nephropathy induced by folic acid and advanced nephropathy induced by doxorubicin. A strong correlation between the proposed biosensor and colorimetric analysis was observed in rat serum samples (*R* ^2^ = 0.988 for ChE; *R* ^2^ = 0.979 for BChE), confirming its reliability and suitability. Biochemical parameter analysis of rat serum samples revealed a positive correlation between total protein levels and ChE/BChE activity, and a negative correlation between C-reactive protein, ALT, and TBK-AP levels and ChE/BChE activity. Furthermore, ChE and BChE activity was closely associated with nephropathy severity markers, including serum creatinine and urea concentrations. Increasing nephropathy severity was associated with a progressive decrease in cholinesterase activity. These findings highlight ChE activity as a potential indicator of nephropathy progression as well as hepatotoxicity and demonstrate the applicability of the proposed choline biosensors as accurate and efficient tools for wide-profile point-of-care biochemical analysis.

## Graphical abstract

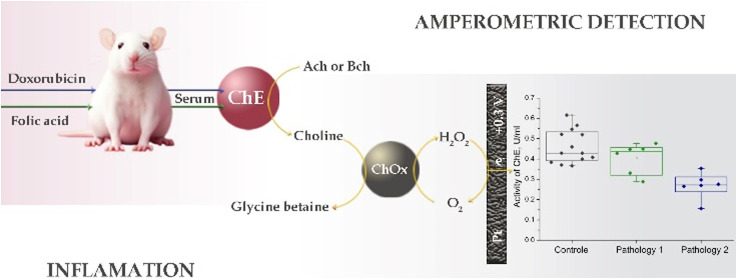

## 1 Introduction

Chronic kidney disease (CKD) is defined by a progressive decline in renal function. Impaired renal clearance in CKD leads to changes in concentrations of numerous metabolites in blood and urine. Also, CKD is not only caused by metabolic imbalances, but there is evidence that it causes an inflammatory response.^1^ For instance, studies have demonstrated that urinary choline and other metabolites of the choline oxidation pathway can serve as independent predictors of CKD progression in patients with type 2 diabetes.^2^ Moreover, serum cholinesterase has been identified as a significant prognostic indicator in patients with nondialysis-dependent CKD. Lower cholinesterase levels were associated with increased all-cause mortality and provided additional predictive value beyond conventional risk models.^3^ Together, these findings highlight the systemic biochemical effects of CKD and support investigating cholinergic biomarkers as potential indicators of disease progression and systemic dysfunction in nephropathy.

Choline levels can rise or fall during inflammatory processes, particularly because it is involved in anti-inflammatory signaling pathways. This relationship makes choline metabolism an area of interest in understanding inflammation-related diseases and developing potential diagnostic tools and therapies. It was demonstrated that choline concentration can indeed increase as esterase activity rises.^4^ Acetylcholine has anti-inflammatory properties; therefore, increased activity of acetylcholinesterase (AChE) and butyrylcholinesterase (BChE) leads to faster acetylcholine breakdown and lower acetylcholine levels. As a result, the anti-inflammatory effect of acetylcholine is reduced or lost, promoting local and systemic inflammation. Thus, higher AChE and BChE activities indirectly indicate decreased acetylcholine levels and increased inflammatory status. On the other hand, uremic toxins that accumulate in the blood due to CKD impact the cholinergic system and inhibit AChE activity.^5,6^ Thus, a decrease in AChE activity may directly reflect CKD progression and the systemic impact of uremic toxins.

Evidently, the cholinergic system and ChE activity play a significant role in CKD. It should be mentioned that CKD is closely associated with hepatic disorders. Patients with CKD require regular monitoring via liver function tests, particularly serum enzyme levels, to manage concomitant liver diseases. Traditionally, hepatotoxicity has been assessed using serum liver biomarkers, including glutamate oxaloacetate transaminase (GOT) and glutamate pyruvate transaminase (GPT), alanine aminotransferase (ALT) and aspartate aminotransferase (AST) and alkaline phosphatase (ALP). However, these biomarkers reflect only late-stage hepatotoxicity and present a significant challenge in laboratory medicine for patients across different stages of CKD. It is well established that ChE, particularly BChE, is synthesized in hepatocytes. Thus, BChE activities can directly reflect liver function and may serve as an early biomarker, as hepatic impairment can lead to decreased serum BchE levels due to reduced synthesis or, conversely, to increased activity resulting from enhanced release from damaged hepatocyte membranes.^7–11^ These studies illustrate the potential of choline, AChE, and BChE as biomarkers for CKD; nevertheless, there is a lack of studies regarding the fluctuations of cholinesterase levels during nephropathy.

Conventional determination of choline and ChE activity relies on spectrophotometric, chromatographic, or mass spectrometry-based methods. Despite the fact that these methodologies provide adequate analytical precision, they frequently necessitate extensive sample preparation, costly instrumentation, and are not well-suited for rapid or point-of-care analysis. Moreover, determining enzymatic activity in complex biological matrices, such as serum, remains challenging due to matrix interference and limited temporal resolution. The use of modern electrochemical biosensors is an effective alternative to conventional methods, as they offer numerous advantages, including ease of use, cost efficiency, portability, sensitivity, and specificity.^12,13^ In order to satisfy the demand for choline monitoring, biosensors with a range of advantages have emerged, including simplicity, high sensitivity, rapid response, and low cost.^14^ Over the last few decades, AChE biosensors have also arisen primarily as a technique for the electrochemical detection of neurotoxic chemicals.^15^ Considerable focus has been placed on the development of straightforward, extremely sensitive, fast, and cost-effective biosensors.^14–16^ Electrochemical biosensing of ChE and BChE activity by amperometric methods typically relies on choline oxidase, which catalyzes the oxidation of choline produced by esterases. During this reaction, hydrogen peroxide is generated as a byproduct and can be readily detected at an electrode surface.^17–19^ Although this sensing strategy is straightforward, its application to complex biological matrices, such as serum, is challenged by interference from endogenous electroactive species that are oxidized at the relatively high potentials required for hydrogen peroxide detection.^20^ This problem can be solved by appropriately selecting enzyme immobilization and electrode protection strategies against interfering substances and by optimizing the biosensor measurement conditions.

A number of choline biosensors have been described in the literature, including our previous study, demonstrating high sensitivity and selectivity toward choline detection. However, only a limited number of these sensors are suitable not only for choline quantification but also for the indirect determination of cholinesterase activity in complex biological matrices. In our recent work, we developed an amperometric choline biosensor based on thermally reduced graphene oxide, which served as an efficient immobilization matrix for choline oxidase (ChOx). Two possible operating mechanisms were demonstrated, depending on the applied working electrode potential: detection via dissolved oxygen consumption at negative potential and via hydrogen peroxide oxidation at positive potentials.^21^

This study assessed a choline biosensor, functioning through a proposed one of mechanism, in rat serum samples to investigate nephrology issues. The operation at a positive electrode potential was selected, relying on the more robust hydrogen peroxide oxidation mechanism. Accordingly, a modified biosensor configuration and a new ChOx immobilization strategy were proposed. In this design, an additional acetylated cellulose layer was introduced as a protective membrane. The sensor was subsequently adapted for the purpose of determining the activity of cholinesterases (AChE and BChE). The biosensor’s application to two experimental nephropathy models (mild nephropathy induced by folic acid and advanced nephropathy induced by doxorubicin) demonstrated the feasibility of monitoring ChE and BChE activities across varying disease severity. These findings were independently confirmed using a conventional colorimetric assay for cholinesterase activity.

While the ChOx/Pt-based biodetection system is well-established, the novelty of this work lies in the integration of an acetyl-modified cellulose membrane into the biosensor construction. This modification effectively reduced the influence of interfering compounds and enabled sensor operation at a lower potential (0.3 V vs Ag/AgCl) compared to conventional choline oxidase-based sensors. Crucially, this system represents one of the first applications of such a biosensor for investigating nephropathy through the detection of changes in cholinesterase activity. Overall, this study highlights cholinesterase activity as a potential biomarker for nephropathy progression and hepatotoxicity, while demonstrating the applicability of the proposed biosensor as an accurate and efficient tool for point-of-care biochemical analysis.

## 2 Materials and Methods

### 2.1 Materials

Choline oxidase from *Alcaligenes sp*. (EC 1.1.3.17, 10.2 U/mg), acetylcholinesterase from *Electrophorus electricus* (EC 3.1.1.7, 441.2 U/mg), butyrylcholinesterase from equine serum (EC 3.1.1.8, 14.6 U/mg) were purchased from Sigma. Choline chloride, acetylcholine chloride, butyrylcholine chloride, serotonin hydrochloride, adenosine 5′-diphosphate, d-glucose, adenosine 5′-diphosphate, uric acid, ascorbic acid, bovine serum albumin (V fraction) (BSA), epinephrine, dopamine hydrochloride, 5,5′-Dithiobis­(2-nitrobenzoic acid) (DTNB), tiocholine iodide, acetylthiocholine, and butyrylthiocholine iodide were obtained from Sigma. Glutaraldehyde 25% was obtained from Merck KGaA. All other chemicals were of analytical grade and used without further purification. Semipermeable polyester (PETE) membrane filters with a thickness of 12 μm and a pore diameter of 0.4 μm were obtained from Sterlitech, USA. Acetylation of the cellulose layer was performed using acetic anhydride and pyridine according to a standard protocol.^22^

### 2.2 Preparation of Biosensor and Electrochemical Measurements

In this study, a ChOx-based enzymatic layer was immobilized on a semipermeable PETE film. Initially, the PETE film was affixed to a rubber O-ring (*d* = 3 mm). The preparation of the enzymatic membrane commenced with the application of 5 μL of a mixture containing 0.05 mg of ChOx from *Alcaligenes sp*., 0.1 mg of BSA, and 0.2 μL of 5% glutaraldehyde in PBS to the inner surface of the film and was immediately covered with a selectivity-providing acetylated cellulose layer. Then, it was incubated at 4 °C overnight to allow membrane formation and optimal enzyme immobilization. Subsequently, the enzymatic membrane was mechanically affixed to the surface of the Pt electrode (Pt/ChOx). The schematic of the constructed biosensor is shown in Figure 1.

**Figure 1:**
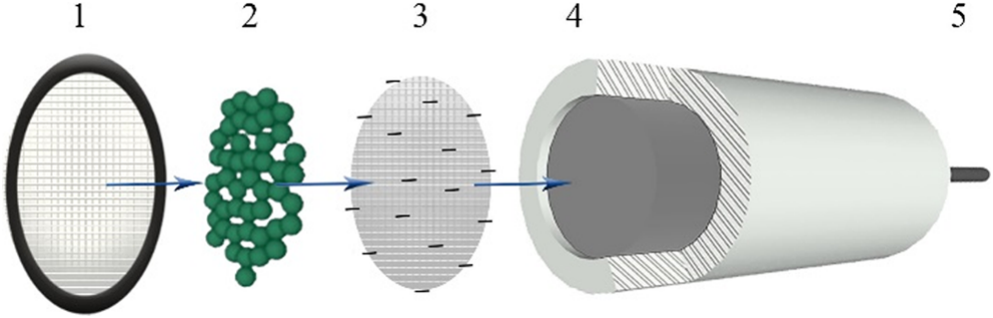
Schematic of the constructed biosensor. One–PETE membrane. Two–Choline oxidase immobilization mixture. Three–Acetylated cellulose layer. Four–Platinum strip (*d* = 3 mm). Five–Electrode body and connection area.

### 2.3 Examination of Biosensor Performance

Chronoamperometric measurements were performed using a conventional three-electrode electrochemical system consisting of an Ag/AgCl reference electrode, a Ti auxiliary electrode, and the biosensor as the working electrode. All measurements were performed in a thermostated 1 mL electrochemical cell at 20 °C with a custom-built potentiostat (Vilnius University, Life Sciences Centre, Institute of Biochemistry). The current–time responses of the biosensor were recorded in a stirred 10 mM PBS with 100 mM KCl, pH 7.2, at an applied potential of 0.3 V vs Ag/AgCl. Biosensor performance was evaluated by recording current–time responses at increasing substrate concentrations. Each measurement was repeated three times, and the mean current response was calculated.

The limit of detection (LOD) was calculated based on the linear regression model described by Shrivastava and Gupta.^23^ According to this model, LOD = 3Sa/b, where Sa is the standard deviation of the response and b is the slope of the calibration curve. In this study, Sa was determined from the standard deviation of the current measured at a selected analyte concentration within the linear calibration range, and b corresponded to the biosensor sensitivity. The values of apparent Michaelis constant (*K*_m_^app^) were determined through the implementation of the electrochemical version of the Michaelis–Menten equation.^24^

The selectivity of the proposed Pt/ChOx choline biosensor was evaluated at 0.3 V vs Ag/AgCl by introducing common interfering compounds, including 0.5 μM dopamine, 0.5 μM adenosine, 0.5 μM serotonin, 1.0 μM uric acid, 25 μM ascorbic acid, 50 μM d-glucose, 70 g/L albumin, 24 μM epinephrine and 240 μM choline into the electrochemical cell.

Analytical recovery was evaluated using a multiple standard addition method. Serum samples from healthy rats (control group) were spiked with choline in the concentration range of 4.9–98 μM. Measurements were performed using 30 μL aliquots of serum in triplicate. Choline concentrations were determined from the calibration curve, and recoveries were calculated according to eq ([Disp-formula eq1]).

$$\mathrm{recovery}\;\left(\%\right)=\left(C_{\mathrm{choline}\;\mathrm{found}}-C_{\mathrm{choline}\;\mathrm{in}\;\mathrm{serum}}\right)/C_{\mathrm{choline}\;\mathrm{added}}\times 100\%$$ 1

### 2.4 Analysis of Choline and Cholinesterase Activity in Biological Samples

To evaluate the effects of the nephropathy process on choline and cholinesterase activity, serum samples from healthy and nephropathy induced rats were analyzed. To evaluate the effects of the nephropathy process on choline and cholinesterase activity, serum samples from rats were analyzed. First, the baseline signal of the choline biosensor was recorded for 20 s, after which 30 μL of undiluted serum was added directly to the electrochemical cell to determine the choline concentration. Following an additional 60 s of signal stabilization, 0.4 mM acetylcholine or butyrylcholine was introduced to assess acetylcholinesterase and butyrylcholinesterase activities, respectively. The biosensor signal rate to acetylcholine or butyrylcholine corresponds to the activity of acetylcholinesterase or butyrylcholinesterase in the serum, respectively.

Additionally, a colorimetric method was applied to determine cholinesterase activity in biological samples. The assays were carried out using a microplate spectrophotometer by monitoring changes in absorbance at 405 nm. In a 96-well microplate, 125 μL of a 3 mM DTNB solution (final concentration 1.875 mM), 25 μL of the sample (prediluted 22-fold), and 25 μL of a 15 mM cholinesterase substrate solution (final concentration 1.875 mM) were mixed. Absorbance changes were recorded over 60 min, and cholinesterase activity was calculated from the absorbance-versus-time curve using its linear region. A calibration curve was constructed using 125 μL of 3 mM DTNB, 25 μL of PBS solution, pH 7.2, and 25 μL of thiocholine solutions at different concentrations (final thiocholine concentrations ranging from 0.3125 to 2.5 mM).

### 2.5 Performance of Nephropathy Models

Nephropathy was induced in rats using two established experimental models. In the first, nephropathy was induced by a single intraperitoneal injection of folic acid (250 mg/kg body weight), following a previously described protocol.^25^ Animals were euthanized on day 7, and blood serum and kidneys were collected for analysis. In the second model, doxorubicin-induced nephropathy was established by intraperitoneal injection of doxorubicin (10 mg/kg, 1 mg/mL in physiological saline)^26^ with samples collected on day 21.

Markers of systemic inflammation and renal function were assessed using commercial diagnostic reagent kits (Filisit Diagnostics, Ukraine). Total protein concentration was determined by the biuret method (Total Protein kit, REF 61900, HP010.01), and C-reactive protein (CRP) levels were measured with a latex-enhanced turbidimetric assay (SRB-TurbiLatex, REF 53705, LA033.07). Alanine aminotransferase (ALT) and aspartate aminotransferase (AST) activities were measured using kinetic kits (ALT KIN, REF 52923, HP001.02; AST KIN, REF 52954, HP004.02). Renal function was evaluated by creatinine and urea concentrations, measured with kinetic photometric (Creatinine KIN, REF 53251, HP014.02) and urease-based enzymatic colorimetric assays (Urease Sechovyna-U, REF 53587, HP018.02), respectively, following the manufacturers’ instructions.

Oxidative stress was assessed by determining thiobarbituric acid–reactive substances (TBARS), based on the reaction with thiobarbituric acid under heating for 15 min, followed by photometric measurement of the colored products at 532 nm.

## 3 Results and Discussion

### 3.1 Characterization of Choline Biosensor

A reagentless amperometric choline biosensor (Pt/ChOx), based on choline oxidase immobilized on a platinum electrode, was developed and operated at a low applied potential of 0.3 V vs Ag/AgCl for the determination of choline and cholinesterase activity. To minimize interference from intercalating compounds, the operating potential was chosen as close to 0 V vs Ag/AgCl as possible without compromising signal sensitivity; therefore, all measurements were performed at 0.3 V vs Ag/AgCl. The buffer solution was set to pH 7.2 to be as close as possible to physiological conditions. This pH ensures applicability of the system not only to serum, but also to urine and saliva samples. Prior to cholinesterase measurements, the biosensor response was evaluated over a range of choline concentrations. The dependence of current density on choline concentration is shown in Figure 2(A).

**Figure 2:**
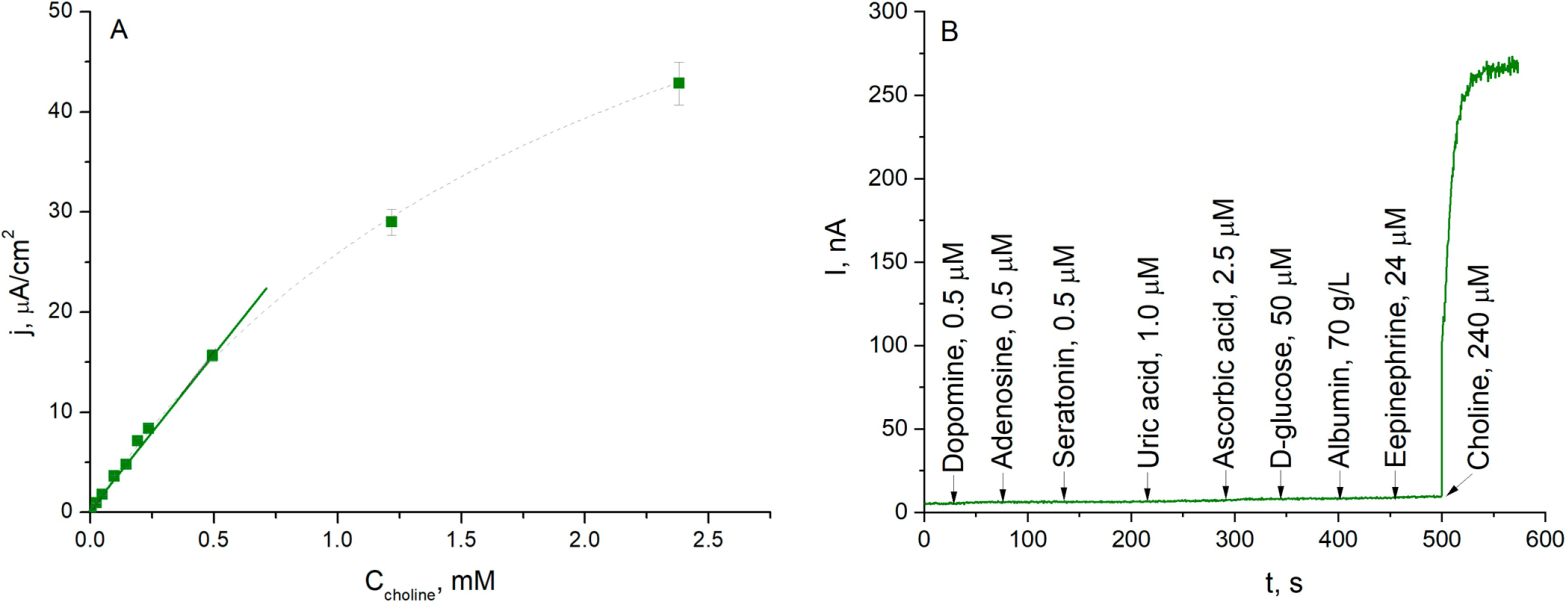
(A)—Dependence of current density on choline concentration for the Pt/ChOx biosensor (solid line, linear fit used to define the linear range; dotted line, Michaelis–Menten fit). (B)—Current–time responses of the Pt/ChOx biosensor in the presence of common interfering compounds.

The Pt/ChOx biosensor demonstrated a linear response to choline within the range of 5–500 μM, with a sensitivity of 32.2 ± 0.3 μA/(mM cm^2^) and a limit of detection (LOD) of 0.67 μM. Its broad linear range makes it suitable for choline determination in various biological samples. The extended linear range of the calibration curve may be attributed to the formation of an effective substrate diffusion barrier within the specially designed biosensor‘s membrane. This interpretation is supported by the measured apparent Michaelis constant (*K*_m_^app^), a key parameter of a biosensor that reflects the degree of linearity of the calibration curves.^24^ Choline oxidase from *Alcaligenes sp*. exhibits a reported Michaelis constant (*K*_m_) of 0.87 mM for choline.^27^ In contrast, the Pt/ChOx demonstrated a substantially higher *K*_m_^app^ = 2.2 ± 0.4 mM. This increase indicates the presence of a significant diffusion barrier between the bulk solution and the enzymatic reaction layer, which restricts substrate accessibility and contributes to the observed broad linear range.

The selectivity of the Pt/ChOx biosensor was evaluated using common biological interferents, including dopamine, adenosine, serotonin, uric acid, ascorbic acid, d-glucose, albumin, epinephrine, and choline (Figure 2(B)). Negligible increases in current (less than 2% relative to choline) were observed for dopamine, uric acid, ascorbic acid, and epinephrine. Previous studies on amperometric cholinesterase biosensors have also shown that negatively charged surfaces, such as Nafion layer, can reduce interference from intercalating compounds.^28^ In Pt/ChOx, Pt electrode surface is protected by a multilayer enzymatic membrane consisting of a PETE layer with a 0.4 μm pore diameter in outer surface and an acetylated cellulose layer in inner side directly adjacent to the Pt surface (Figure 1.). These membranes serve a dual function: They physically shield the enzymatic layer and electrode surface by restricting the infiltration of large molecules from the external environment, thereby minimizing nonspecific protein adsorption and electrode fouling. Meanwhile, the negative charge generated by the acetyl groups at the electrode interface utilizes electrostatic repulsion to deflect small negatively charged molecules, such as ascorbic acid, while capturing positively charged molecules, such as dopamine. This layered structure significantly reduces or even eliminates nonspecific reactions on the Pt surface. Moreover, this multilayer architecture prevents ChOx layer leaching during use.

In order to adapt the biosensor for the determination of cholinesterase activity in rat serum, enabling its use as a biomarker for monitoring the dynamics of inflammatory processes, the analytical recovery of the Pt/ChOx biosensor was evaluated using the multiple-spike standard addition method. The results, including the added and found choline concentrations, standard deviation, and recovery, are shown in Table 1.

**Table 1:** Analytical Performance of the Pt/ChOx Biosensor in Rat Serum Samples (*n* = 3)

C (choline added), μM	C (choline found), μM	recovery, %	RSD, %
0	100.1 ± 0.1		
4.9	105.0 ± 0.1	101	3.6
25	125.2 ± 0.2	100	1.4
50	147.5 ± 0.5	95	1.9
98	196.3 ± 0.7	98	1.3

The Pt/ChOx biosensor demonstrated good analytical recovery, with recovery rates ranging from 95 to 101% and RSD of 1.3–3.6% in healthy rats serum. A slight increase in the biosensor signal was observed at low added choline concentrations, which may be attributed to the presence of interfering compounds, such as ascorbic acid.

Since the biosensor performance depends on choline oxidase stability, operational and storage stability were evaluated using a fixed choline concentration. It was found that over 30 consecutive measurements performed throughout an 8-h working day, the maximum deviation was about 4%. The biosensor was stored at room temperature for 28 days, during which its activity decreased to 88%. These results demonstrate that the constructed biosensor exhibits both excellent operational and storage stability. In contrast, the long-term storage stability was assessed by maintaining the membrane with immobilized ChOx in dry conditions at +4 °C. Following a 7-month storage period, the membrane was attached to the Pt electrode for testing. The measurements revealed that the biosensor lost approximately 93% of its initial sensitivity. These results suggest that prolonged dry storage damages the tertiary structure of ChOx and consequently, the sensitivity of the biosensor decreases dramatically. This suggests that immobilization method of ChOx is very effective, but the storage requires a aqueous environment. In summary, the proposed multilayer membrane architecture and biosensor design ensure high selectivity and robust operational stability under wet storage conditions.

### 3.2 Evaluation of Esterase Activity by Pt/ChOx

#### 3.2.1 Mechanism of Cholinesterase Activity Determination

After evaluating the performance of the Pt/ChOx biosensor and determining its main analytical characteristics at 0.3 V vs Ag/AgCl, the biosensor was further adapted for the determination of AChE and BChE activity in rat serum samples. Before experiments with cholinesterases (acetylcholinesterase (AChE) and butyrylcholinesterase (BChE)), the biosensor response to the esterases substrates used—acetylcholine (ACh) and butyrylcholine (BCh)—was investigated. It was determined that ACh and BCh generated small anodic currents of 2.2 ± 0.2 nA and 12.0 ± 0.3 nA, respectively, at a substrate concentration of 0.4 mM. These current changes are negligible, as an equivalent concentration of choline generates an anodic current of approximately 280 ± 3 nA. The biosensor response to ACh and BCh may arise from the spontaneous hydrolysis of these substrates in aqueous solutions. However, compared to cholinesterase-catalyzed hydrolysis, spontaneous hydrolysis is a significantly slower process.^29,30^ To minimize the influence of spontaneous hydrolysis, fresh ACh and BCh solutions were prepared daily. Another possible cause of a slight change in anodic current is a nonspecific reaction. Considering that the response to ACh and BCh is small, they will not have a significant impact in further studies.

Knowing that the Pt/ChOx biosensor operates properly, it was applied for the evaluation of AChE and BChE activity. The principal scheme of the Pt/ChOx biosensor action mechanism in the presence of esterases is presented in Figure 3.

**Figure 3:**
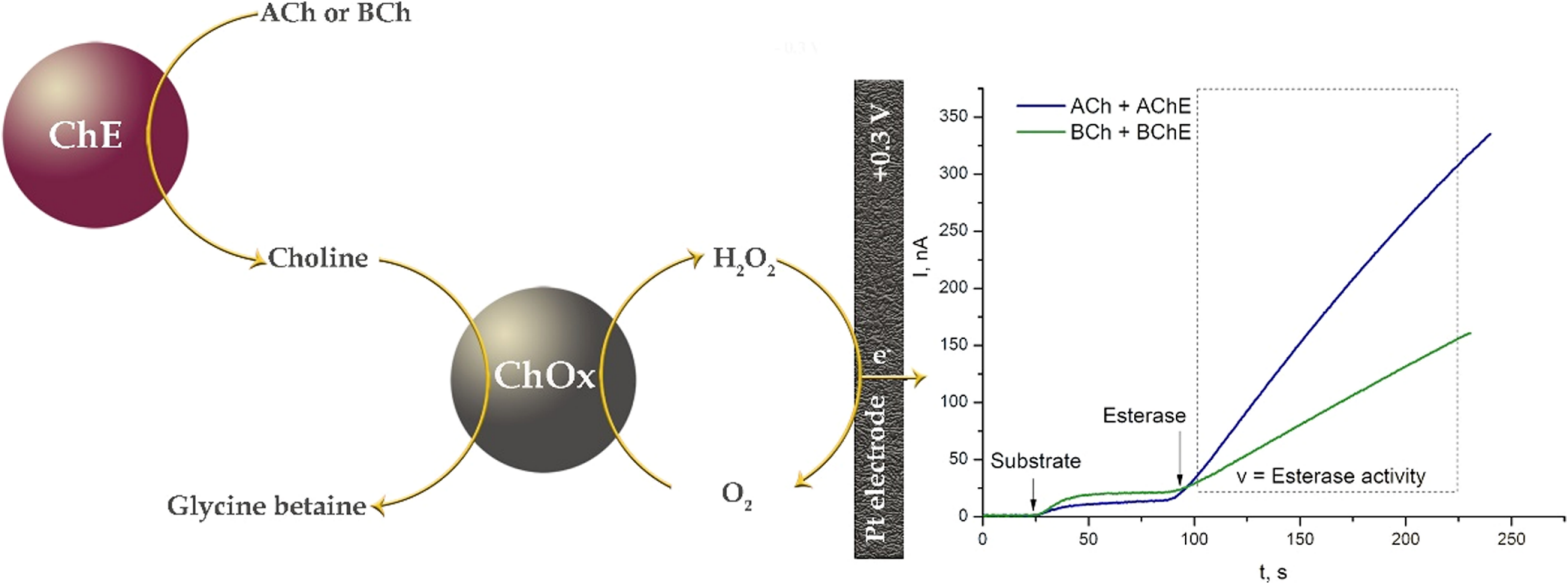
Principle of detecting cholinesterase activity based on the amperometric action of the choline biosensor and its current–time responses.

At the 20th second of biosensor operation, 0.4 mM ACh or BCh solution was added to the electrochemical cell (the concentration of 0.4 mM ACh and BCh was determined to be optimal during measurements performed with a constant enzyme concentration of 0.5 U/ml AChE or BChE in the solution, data not shown), and the increase in anodic current generated by the Pt/ChOx biosensor (approximately 60 s) was recorded. After 80 s of measurement, 5–50 μL of 10 U/ml AChE or BChE was added to the electrochemical cell, and the anodic current response generated by the biosensor was monitored for an additional 150 s. The rate of development of this response reflects the activity of the corresponding cholinesterase.

Electrochemical signals obtained at different amounts of AChE and BChE using ACh or BCh as substrates are presented in Figure 4, panels B, C, E, and F. Calibration curves derived from these data are shown in Figure 4, panels A and D, based on the dependence of the current change rate on the added amount of cholinesterase. The biosensor’s sensitivity was calculated from the slopes of these curves, where the current change rate (nA/s) was plotted on the *Y*-axis against the enzyme activity concentration (U/ml) on the *X*-axis. This approach yielded a sensitivity unit of nA ml/(s U), and the resulting sensitivity values are summarized in Table 2.

**Figure 4:**
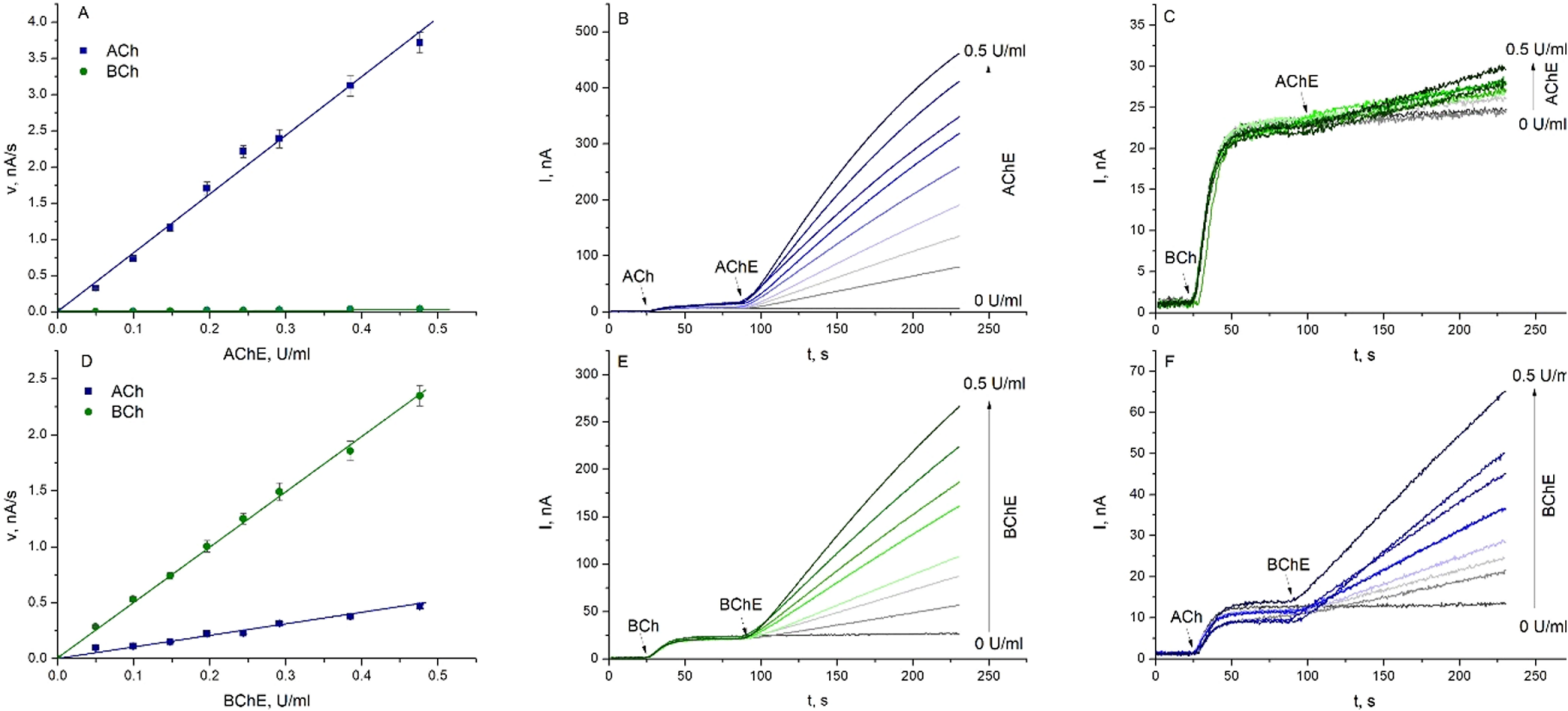
Calibration curves of the Pt/ChOx biosensor for different amounts of cholinesterase. (A)—AChE, (D)—BChE. Real electrochemical responses obtained at different concentrations of cholinesterases. (B)—AChE with ACh as substrate, (C)—AChE with BCh as substrate, (E)—BChE with BCh as substrate, and (F)—BChE with ACh as substrate.

**Table 2:** Comparison of Characteristics of Amperometric Biosensors for Assessing Cholinesterase Activity

	analyte	substrate	E, V vs Ag/AgCl	LOD, U/ml	linear range, U/ml	stability	sensitivity, nA ml/(s U)	refs
Pt/ChO/PA	AChE	ACh	0.65	NA	0.0001–0.01	NA	16.7^a^	^31^
	BChE	BCh			0.00003–0.003		13.3^a^	
GC/lmChO/Nafion	BChE	ACh	1.0	0.002	up to 2	4 weeks	48.5	^28^
Pt/oPPy/ChO	BChE	BTCh	0.656	0.0005	Up to 0.6	4 weeks (4 °C, remaining activity 70%)	42.9	^17^
	BChE	ACh			Up to 0.6		NA	
	BChE	BCh			Up to 0.18		NA	
	AChE	ACh			Up to 0.2		NA	
GC/NNO	AChE	ACh	0.6	0.0141	0.05–2	NA	8.56	^32^
Pt/ChO	AChE	ACh	0.3	0.003	Up to 0.5	4 weeks (R.T., remaining activity 88%)	8.07	This work
	AChE	BCh		Negligible	Up to 0.5		0.09	
	BChE	ACh		0.025	Up to 0.5		0.94	
	BChE	BCh		0.005	Up to 0.5		4.87	

a Data calculated from the graph in the figure. PA—polyamide, GC—glassy carbon electrode, lmChO—lipid-modified choline oxidase, oPPy—overoxidized polypyrrole, NNO—nortropine-N-oxyl.

From the data above, it is evident that AChE efficiently catalyzes the hydrolysis of acetylcholine, whereas its activity toward butyrylcholine is negligible. In contrast, BchE catalyzes the hydrolysis of both ACh and BCh. It is worth noting that hydrolysis of the natural BChE substrate, BCh, occurs approximately five times more efficiently than that of ACh.Therefore, when ACh is used as the substrate, both AChE and BChE activities are measured, whereas using butyrylcholine allows selective determination of BChE activity. To demonstrate the sensor’s response stability and reliability, five consecutive measurements of 0.3 U/ml AChE and BChE were performed, yielding relative standard deviations (RSD) of 0.19% and 0.23%, respectively. These results demonstrate excellent biosensor reproducibility in ChE activity measurements. The limits of detection (LOD) for AChE and BChE were also determined using their optimal substrates, ACh and BCh, respectively, with values of 0.003 U/ml and 0.005 U/ml.

Compared with similar sensors, the constructed biosensor exhibits lower sensitivity but better stability and the ability to be stored at room temperature (Table 2).

### 3.3 Studies of Esterase Activity in Biological Samples of Model Animals

To validate proposed biosensor performance in rat blood serum samples, two experimental nephropathy models of rats were investigated and analyzed: Mild nephropathy induced by folic acid (N1) and advanced nephropathy induced by doxorubicin (N2). To test the hypothesis that choline levels and cholinesterase activities in serum change depending on the nature of inflammation, an analysis of variance (ANOVA) with the Tukey post hoc test was performed. The dependence of choline concentration and cholinesterase activity on the type of inflammation in blood serum is presented in the quartile plots in Figure 5(C).

**Figure 5:**
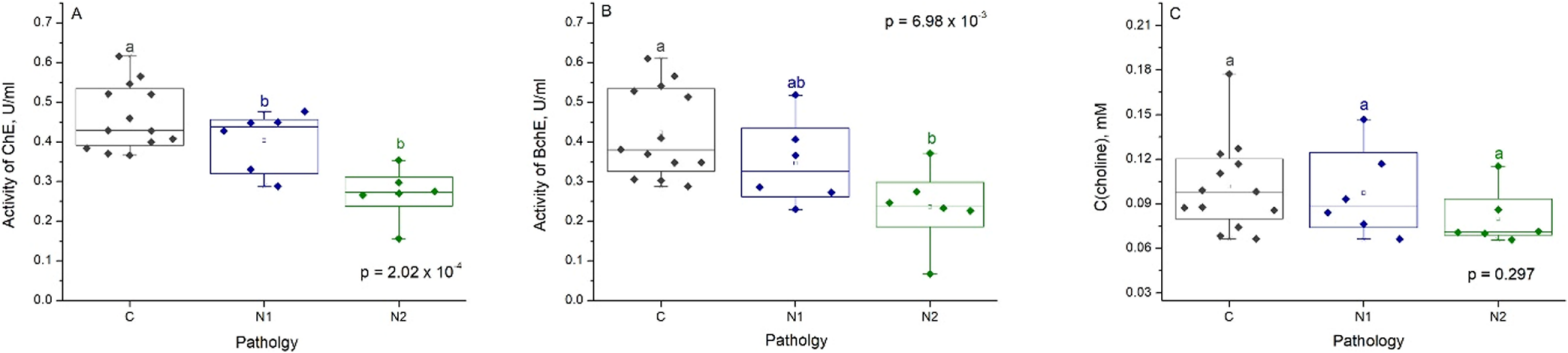
Dependence of total cholinesterase, BChE activities, and choline levels on the type of inflammation in blood serum: (A)—total cholinesterase, (B)—BChE activity, (C)—choline in Control (C), nephropathy (N1), and deeper nephropathy (N2) groups. Data were obtained using a Pt/ChOx biosensor (*E* = 0.3 V vs Ag/AgCl, 10 mM PBS, pH 7.2). Statistically significant differences between groups are indicated by different letters (one-way ANOVA with Tukey’s post hoc test, *p* < 0.05).

The choline concentration determined using the biosensor ranged from 0.07 to 0.18 mM, and a slight decrease was observed in the pathological group compared with the control group; however, the overall ANOVA *p*-value was 0.297. This p-value, which is higher than 0.05, indicates that there is no statistically significant difference among the groups. In this rat model of induced inflammation, changes in cholinesterase activity were expected, which would consequently lead to changes in choline levels in the organism. However, analysis of rat serum samples using the constructed biosensor did not reveal significant differences in choline levels between healthy and patological rats. Therefore, to further test our hypothesis that inflammatory processes in rats affect cholinesterase activity, cholinesterase activity in serum samples was investigated. Meanwhile, analysis of esterase activity in the samples revealed that ChE (AChE + BChE) and BChE groups were statistically different (Figure 5(A,B)). The determined p-values were 2.02 × 10^–4^ and 6.98 × 10^–3^ for the ChE and BChE groups, respectively, which were significantly lower than the chosen significance level of 0.05. To determine which group means differed significantly, the Tukey posthoc test was applied. Statistical analysis of total ChE and BChE in serum revealed that the means of the N2 groups differed significantly from those of the control group (C) in both cases. Moreover, a difference between the N1 and N2 groups was observed in the ChE case. No significant differences were observed between the N1 and the control, indicating statistical similarity among these groups.

To confirm the reliability of the electrochemical (Pt/ChOx-based) method, biological samples were also analyzed using a colorimetric assay. Colorimetric measurements were performed using acetylthiocholine or butyrylthiocholine as substrates for cholinesterases. The correlations between ChE and BChE activity values determined using Pt/ChOx and the colorimetric method are presented in the Figure 6.

**Figure 6:**
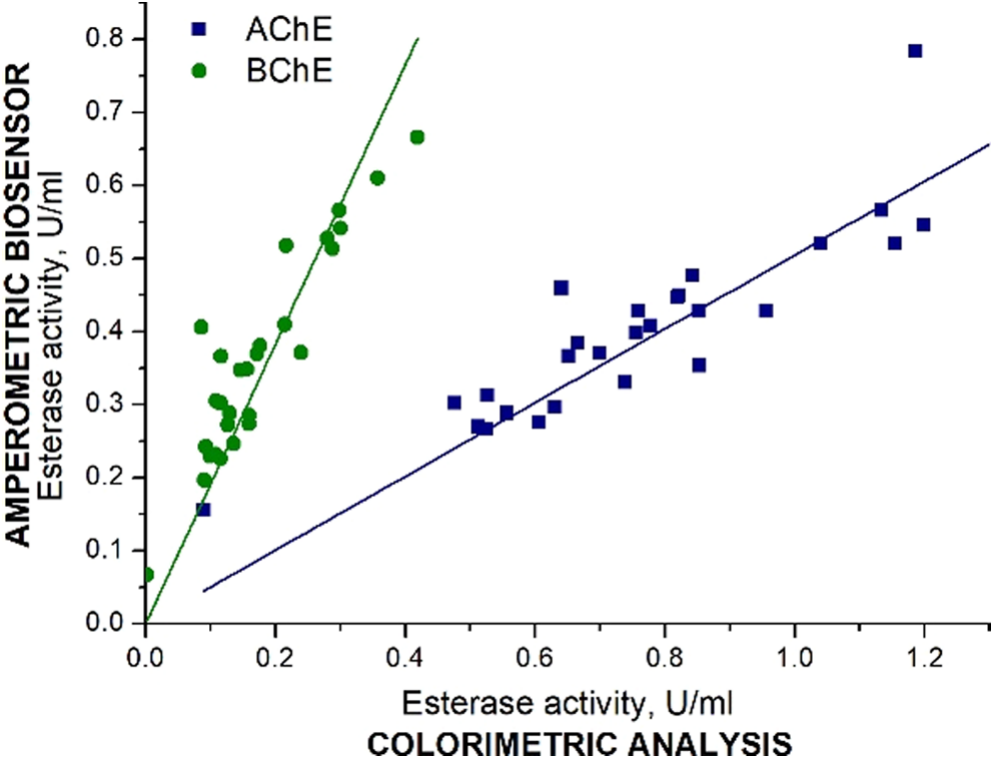
Correlation between ChE and BChE activities determined by Pt/ChOx and colorimetric methods.

As can be seen from the data above, the relationship between the results obtained using the biosensor and those from the colorimetric analysis is linear, with correlation coefficients of 0.988 and 0.979 for AChE and BChE, respectively. These high correlation coefficients indicate excellent agreement between the two analytical methods. In addition, the regression lines intersect the *Y*-axis at zero, indicating the absence of systematic bias between the methods and demonstrating that both methods exhibit a consistent zero response.

### 3.4 Comparison of Esterase Activity with Biological Inflammatory Parameters

To evaluate whether folic acid (N1) and doxorubicin (N2) induced nephropathy, standard blood tests were performed in rats to measure urea, creatinine, total protein (TP), C-reactive protein (CRP), alanine aminotransferase (ALT), aspartate aminotransferase (AST) and TBK-active products (TBK-AP) were determined by the reaction with thiobarbituric acid (TBK). These parameters reflected both the presence and severity of nephropathy. As anticipated, impaired renal filtration led to increased blood creatinine and urea levels in both N1 and N2 groups, with higher elevations in N2 rats, reflecting more severe nephropathy (Figure 7(A,B)). Total protein was assessed as a marker of proteinuria, which contributes to kidney damage and predicts CKD progression^33^ (Figure 7(C)). CRP levels confirmed the presence of systemic inflammation in both models (Figure 7(D)). Overall, these biochemical data indicate that induction with folic acid and doxorubicin successfully induced nephropathy of varying severity in the experimental rats. It should be noted that CKD is accompanied by systemic low-grade inflammation, oxidative stress, and progressive protein metabolism disorders. A decrease in total protein concentration in blood serum against a background of pronounced proteinuria is combined with a significant increase in C-reactive protein (CRP)^34,35^ and TBK-active products (markers of lipid peroxidation)^36,37^ while transaminase activity (ALT, AST) is usually reduced.^38^ These changes reflect both the degree of renal dysfunction and systemic inflammatory-metabolic imbalance, making them important biomarkers of nephropathy progression.

**Figure 7:**
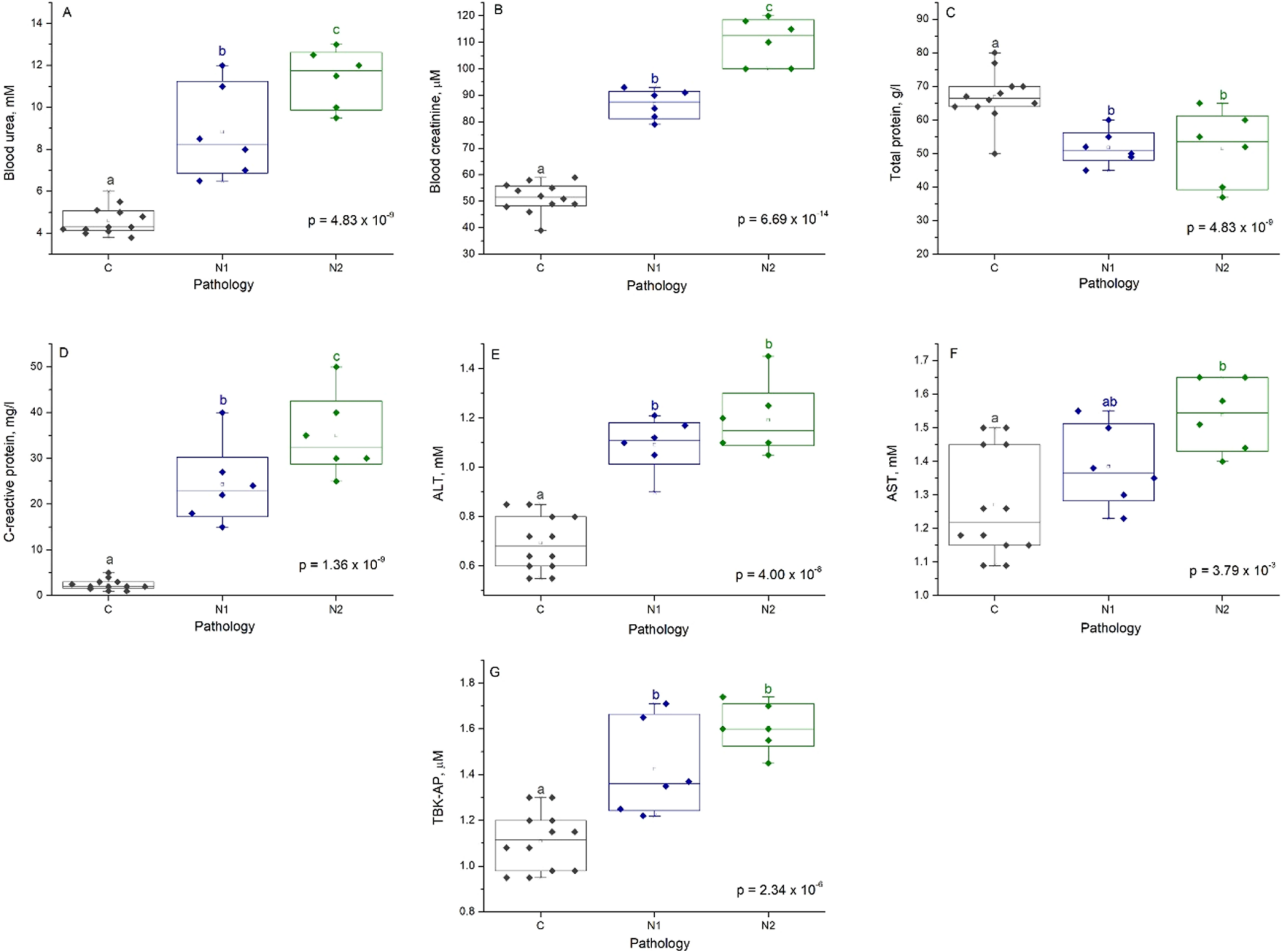
Blood parameters of rats with folic acid (N1) and doxorubicin (N2) induced nephropathy. (A)—blood urea level. (B)—blood creatinine level. (C)—total amount of protein in blood. (D)—amount of C-reactive protein in blood. (E)—alanine aminotransferase (ALT). (F)—aspartate aminotransferase (AST). (G) —TBK-active products (TBK-AP) were determined by the reaction with thiobarbituric acid (TBK). Statistically significant differences between groups are indicated by different letters (one-way ANOVA with Tukey’s post hoc test, *p* < 0.05).

When comparing the relationships between the biochemical parameter values presented in Figure 7 and nephropathy severity, it can be observed that, in all cases except total protein, the parameter values increase with increasing nephropathy severity. In contrast, TP levels decrease as nephropathy severity increases, similarly to the trends observed for the measured ChE and BChE activities.

ANOVA analysis revealed that all biochemical parameter groups differed significantly (*p* < 0.05). Tukey’s post hoc test indicated that blood creatinine, urea, and CRP are the biomarkers that best reflect nephropathy severity, since all group comparisons were significantly different (C–N1, C–N2, and N1–N2). For other parameters, such as TBK-AP, ALT, and TP, significant differences were observed between the C–N1 and C–N2 groups, similar to the ChE results. For AST, only the N2 group differed significantly from the control group.

Comparing the obtained biochemical parameters with the determined ChE values (Figures 5 and 7) revealed a moderate positive Pearson correlation between total protein levels and ChE activity (*r* = 0.42). In contrast, significant negative correlations were observed between ChE activity and CRP (*r* = −0.73), ALT (*r* = −0.60), and TBK-AP (*r* = −0.66). BChE activity showed weaker correlations with the same biochemical parameters. Nevertheless, both ChE and BChE activities were strongly associated with markers of nephropathy severity, including blood urea and creatinine concentrations (Pearson *r* = −0.76 and −0.63 for urea, and −0.73 and −0.61 for creatinine, respectively). Increasing nephropathy severity was accompanied by a progressive decrease in cholinesterase activity. Given that creatinine and urea act as uremic toxins,^5,6^ it can be concluded that they can inhibit AChE activity during various stages of nephropathy; consequently, the overall ChE activity is reduced as well. Meanwhile, serum ChE, particularly BChE, is synthesized in hepatocytes; therefore, ChE and BChE activity can directly reflect liver function. Traditionally, liver function has been assessed using serum biomarkers such as ALT and AST. However, these biomarkers primarily indicate cellular damage that has already occurred, representing a relatively late stage of hepatotoxicity^11^. Based on the strong correlations observed with these biochemical parameters, described in this study, we may suggest that the nephropathy severity-dependent decrease in ChE and BChE activity could serve as a valuable biomarker of clear impaired liver function and as a simple tool for investigating conditions associated with hepatic dysfunction. Our results demonstrated that the selected compounds induced not only renal impairment but also likely affected liver function. These findings suggest that serum cholinesterase activity may serve as a potential complementary biomarker for both liver and kidney function. Such effects are not unexpected, as numerous studies have reported a functional interplay between the liver and kidneys, indicating a synergistic relationship between these organs.^39–41^ Overall, these findings highlight cholinesterase activity as a potential indicator of nephropathy progression and hepatotoxicity. The results also demonstrate the applicability of the proposed choline biosensor as an accurate and efficient tool for point-of-care biochemical analysis.

To illustrate the practical adoption of the proposed biosensor, a point-of-care clinical scenario is envisioned. In this workflow, the assessment is performed using a semiautomated analyzer integrating the developed biosensor. A fixed volume of patient serum is introduced into the electrochemical cell, where the system, controlled by a compact potentiostat, records the current response at 0.3 V vs Ag/AgCl. Following the measurement, an integrated automatic washing system cleans the cell to prevent cross-contamination, ensuring the device is ready for the next sample within minutes. For patients with chronic kidney disease, such a device could be utilized for routine nephropathy assessment at the bedside or even for home-monitoring. A significant shift in the detected enzyme activity would serve as an early warning signal for disease progression or drug-induced hepatotoxicity, allowing clinicians to adjust treatment protocols promptly without the delays associated with centralized laboratory results. Such a scenario is feasible due to the high stability of the biosensor at room temperature.

## 4 Conclusions

This study demonstrates the successful development and application of an amperometric choline biosensor (Pt/ChOx) for the determination of choline and cholinesterase (AChE and BChE) activity in serum samples from healthy and nephropathy induced rats. The biosensor exhibited a broad linear response range of 5–500 μM for choline with a sensitivity of 32.2 ± 0.3 μA/(mM cm^2^), and minimal interference from common biological compounds, confirming its suitability for complex biological samples. The biosensor was also adaptedfor the determination of AChE and BChE determination, achieving LODs of 0.003 and 0.005 U/ml, respectively, with a linear range up to 0.5 U/ml. Notably, the biosensor retains a 88% of its initial sensitivity after 28 days of use. The Pt/ChOx biosensor efficacy to measure AChE and BChE activities was controlled by the choice of substrate (acetylcholine for total ChE, butyrylcholine for BChE). Validation against a conventional colorimetric assay demonstrated strong correlations (*R* ^2^ = 0.988 for ChE, *R* ^2^ = 0.979 for BChE).

Results obtained by investigating using Pt/ChOx two experimental nephropathy models (mild nephropathy induced by folic acid and advanced nephropathy induced by doxorubicin) revealed a progressive decrease in ChE and BChE activities with increasing disease severity. These changes were investigated in terms of correlation with standard biochemical markers of nephropathy, including creatinine, urea, total protein, and C-reactive protein, and it was concluded that cholinesterase activity reflects both renal dysfunction and systemic inflammatory status.

Overall, these findings highlight cholinesterase activity as a sensitive biochemical indicator of nephropathy progression—associated with uremic toxins—as well as hepatotoxicity. Furthermore, they demonstrate that the developed Pt/ChOx choline biosensor offers great promise as a versatile, point-of-care compatible instrument for tracking cholinergic indicators in biological specimens.
